# Building Test Batteries Based on Analyzing Random Number Generator Tests within the Framework of Algorithmic Information Theory

**DOI:** 10.3390/e26060513

**Published:** 2024-06-14

**Authors:** Boris Ryabko

**Affiliations:** 1 Federal Research Center for Information and Computational Technologies, Novosibirsk 630090, Russia; boris@ryabko.net; 2 Institute of Informatics and Computer Engineering, Siberian State University of Telecommunications and Informatics, Novosibirsk 630102, Russia

**Keywords:** random number generator, statistical test, algorithmic information theory, battery of tests, data compression, universal coding, Hausdorff dimension

## Abstract

The problem of testing random number generators is considered and a new method for comparing the power of different statistical tests is proposed. It is based on the definitions of random sequence developed in the framework of algorithmic information theory and allows comparing the power of different tests in some cases when the available methods of mathematical statistics do not distinguish between tests. In particular, it is shown that tests based on data compression methods using dictionaries should be included in test batteries.

## 1. Introduction

Random numbers play an important role in cryptography, gambling, Monte Carlo methods and many other applications. Nowadays, random numbers are generated using so-called random number generators (RNGs), and the “quality” of the generated numbers is evaluated using special statistical tests [1]. This problem is so important for applications that there are special standards for RNGs and for so-called test batteries, that is, sets of tests. The current practice for using an RNG is to verify the sequences it generates with tests from some battery (such as those recommended by [2,3] or other standards).

Many statistical tests are designed to test some deviations from randomness described as classes of random processes (e.g., Bernoulli process with unequal probabilities 0 and 1, Markov chains with some unknown parameters, stationary ergodic processes, etc.) [1,2,3,4,5].

A natural question is: how do we compare different tests and, in particular, create a suitable battery of tests? Currently, this question is mostly addressed experimentally: possible candidate tests are applied to a set of known RNGs and the tests that reject more (“bad”) RNGs are suitable candidates for the battery. In addition, researchers try to choose independent tests (i.e., those that reject different RNGs) and take into account other natural properties (e.g., testing speed, etc.) [1,2,3,4]. Obviously, such an approach depends significantly on the set of selected tests and RNGs pre-selected for consideration. It is worth noting that at present there are dozens of RNGs and tests, and their number is growing fast, so the recommended batteries of tests are rather unstable (see [4]).

It is clear that increasing the number of tests in a battery increases the total testing time or, conversely, if testing time is limited, increasing the number of tests causes the length of the binary sequence being examined to decrease and therefore the power of any battery test is reduced. Therefore, it is highly desirable to include in the battery powerful tests designed for different deviations from randomness.

The goal of this paper is to develop a theoretical framework for test comparison and illustrate it by comparing some popular tests. The main idea of the proposed approach is based on the definition of randomness developed in algorithmic information theory (AIT). Apparently, it is natural to use this theory, since it is the only mathematically correct theory that formally defines what a random binary sequence is, and by definition any RNG should generate such sequences. Similar to AIT, we extend the notion of “random sequence” to any statistical test *T*, and then compare the “size” of the set of random sequences corresponding to different tests. More precisely, let $R_{T_{1}}$ and $R_{T_{2}}$ be random sequences according to $T_{1}$ and $T_{2}$. Then, if $\dim(R_{T_{1}}\setminus R_{T_{2}})$ >0, then $T_{1}$ accepts a large set of sequences as random, whereas $T_{2}$ rejects these sequences as non-random. So, in this sense, a $T_{1}$ test cannot replace $T_{2}$ in a battery of tests (here dim is the Hausdorff dimension.).

Based on this approach, we give some practical recommendations for building test batteries. In particular, we recommend including in the test batteries a test based on a dictionary data compressor, like Lempel–Ziv codes [6], grammar-based codes [7] and some others.

The rest of this paper consists is organized as follows. The next part contains definitions and preliminary information, the third part is a comparison of the test performance on Markov processes with different memories and general stationary processes, and the fourth part investigates tests based on Lempel–Ziv data compressors. The fifth part is a brief conclusion; some of the concepts used in this paper are given in the Appendix A.

## 2. Definitions and Preliminaries

### 2.1. Hypothesis Testing

In hypothesis testing, there is a main hypothesis $H_{0}=\;\{$the sequence *x* is random} and an alternative hypothesis $H_{1}=\neg H_{0}$. (In the probabilistic approach, $H_{0}$ is that the sequence is generated by a Bernoulli source with equal probabilities 0 and 1.) A test is an algorithm for which the input is the prefix $x_{1}\ldots x_{n}$ (of the infinite sequence $x_{1},\ldots ,x_{n},\ldots$) and the output is one of two possible words: *random* or *non-random* (meaning that the sequence is random or non-random, respectively).

Let there be a hypothesis $H_{0}$, some alternative $H_{1}$, let *T* be a test and τ be a statistic, that is, a function on ${\left\{0,1\right\}}^{n}$ which is applied to a binary sequence $x=x_{1}\ldots x_{n}$. Here and below ${\left\{0,1\right\}}^{n}$ is the set of all *n*-bit binary words, ${\left\{0,1\right\}}^{\infty }$ is the set of all infinite words $x_{1}x_{2}\ldots ,x_{i}\in \left\{0,1\right\}$.

By definition, Type I error occurs if $H_{0}$ is true and $H_{0}$ is rejected; the significance level is defined as the probability of the Type I error. Denote the critical region of the test *T* for the significance level α by ${\bar{C}}_{T}\left(\alpha ,n\right)$ and let $C_{T}\left(\alpha ,n\right)$$={\left\{0,1\right\}}^{n}\setminus {\bar{C}}_{T}\left(\alpha ,n\right).$ Recall that, by definition, $H_{0}$ is rejected if and only if $x\in {\bar{C}}_{T}\left(\alpha ,n\right)$ and, hence,

(1) $$|{\bar{C}}_{T}\left(\alpha ,n\right)|\leq 2^{n}\alpha \;,$$

see [8]. We also apply another natural limitation. We consider only tests *T* such that for all *n* and $\alpha_{1}<\alpha_{2}$$\;\;\;{\bar{C}}_{T}\left(\alpha_{1},n\right)\subset {\bar{C}}_{T}\left(\alpha_{2},n\right)$. (Here and below, |X| is the number of elements *X* if *X* is a set, and the length of *X*, if *X* is a word.)

A finite sequence $x_{1}\ldots x_{n}$ is considered random for a given test *T* and the significance level α if it belongs to $C_{T}\left(\alpha ,n\right)$.

### 2.2. Batteries of Tests

Let us consider a situation where the randomness testing is performed by conducting a battery of statistical tests for randomness. Suppose that the battery $\hat{T}$ contains a finite or countable set of tests $T_{1},T_{2},\ldots$ and $\alpha_{i}$ is the significance level of *i*-th test, i=1,2,…. If the battery is applied in such a way that the hypothesis $H_{0}$ is rejected when at least one test in the battery rejects it, then the significance level α of this battery satisfies the following inequality:

(2) $$\alpha \leq \sum_{i=1}^{\infty }\alpha_{i}\;,$$

because P(A+B)≤P(A)+P(B) for any events *A* and *B* (This inequality is a simple extension of the so-called Bonferroni correction, see [9]).

It will be convenient to formulate this inequality in a different way. Suppose there is some α∈(0,1) and a sequence ω of non-negative $\omega_{i}$ such that $\sum_{i=1}^{\infty }\omega_{i}\leq 1$. For example, we can define the following sequence $\omega^{*}$:

(3) $$\omega_{i}^{*}=1/\left(i\left(i+1\right)\right)\;\;\;\;i=1,2,\ldots \;\;.$$

If the significance level $T_{i}$ equals $\alpha \omega_{i}$, then the significance level of the battery $\hat{T}$ is not grater than α. (Indeed, from (2) we obtain $\sum_{i=1}\alpha_{i}=\sum_{i=1}\left(\alpha \omega_{i}\right)$$=\alpha \sum_{i=1}\omega_{i}\leq \alpha$.) Note that this simple observation makes it possible to treat a test battery as a single test.

#### 2.2.1. Random and Non-Random Infinite Sequences

Kolmogorov complexity is one of the central notations of algorithmic information theory (AIT), see [10,11,12,13,14,15,16,17,18]. We will consider the so-called prefix-free Kolmogorov complexity K(u), which is defined on finite binary words *u* and is closely related to the notion of randomness. More precisely, an infinite binary sequence $x=x_{1}x_{2}\ldots$ is random if there exists a constant *C* such that

(4) $$n-K(x_{1}\ldots x_{n})<C$$

for all *n*, see [19]. Conversely, the sequence *x* is non-random if

$$\forall \;\;C>0\;\;\exists \;n_{C}\;\;\;n_{C}-K\left(x_{1}\ldots x_{n_{C}}\right)\geq C$$

In some sense, Kolmogorov complexity is the length of the shortest lossless prefix-free code, that is, for any (algorithmically realisable) code *f* there exists a constant $c_{f}$ for which $K\left(u\right)\leq \left|f\left(u\right)\right|+c_{f}$ [10,11,12,13,14,15,16]. Recall that a code *f* is lossless if there is a mapping $f^{-1}$ such that for any word *u*$\;f^{-1}\left(f\left(u\right)\right)=u$ and *f* is prefix-free (or unprefixed) if for any words u,v, f(u) is not a prefix of f(v) and f(v) is not a prefix of f(u).

Let *f* be a lossless prefix-free code defined for all finite words. Similarly to (4), we call it random with respect to *f* if there is a constant $C_{f}$ such that

(5) $$n-|f\left(x_{1}\ldots x_{n}\right)|\;<C_{f}$$

for all *n*. We call this difference the statistic corresponding to *f* and define

(6) $$\tau_{f}\left(x_{1}\ldots x_{n}\right)=n-\left|f\left(x_{1}\ldots x_{n}\right)\right|.$$

Similarly, the sequence *x* is non-random with respect to *f* if

(7) $$\forall C>0\;\;\;\exists \;n_{C}\;\;\;n_{C}-|f(x_{1}\ldots x_{n_{C}}|\;\geq C\;.$$

Informally, *x* is random with respect to *f* if the statistic $\tau_{f}$ is bounded by some constant on all prefixes $x_{1}\ldots x_{n}$ and, conversely, *x* is non-random if $\tau_{f}$ is unbounded when the prefix length grows.

Based on these definitions, we can reformulate the concepts of randomness and non-randomness in a manner similar to what is customary in mathematical statistics. Namely, for any α∈(0,1) we define the set $\left\{y=y_{1}\ldots y_{n}:\tau_{f}\left(y\right)\geq -\log\alpha \right\}$. It is easy to see that (1) is valid and, therefore, this set represents the critical region ${\bar{C}}_{T}\left(\alpha ,n\right)$, where the test *T* is as follows: $T=\{x_{1}\ldots x_{n}$: $\tau_{f}\left(x_{1}\ldots x_{n}\right)<\alpha \}$.

Based on these consideration, (6) and the definitions of randomness (4), (5) we give the following definition of randomness and non-randomness for the statistic $\tau_{f}$ and corresponding test $T_{f}$. An infinite sequence $x=x_{1}x_{2}\ldots$ is random according to the test $T_{f}$ if there exists such α>0 that for any integer *n* and this α the word $x_{1}\ldots x_{n}$ is random (according to the $T_{f}$ test). Otherwise, the sequence *x* is non-random.

Note that we can use the statistic

$$\tau_{f}=n-\left|f\left(x_{1}\ldots x_{n}\right)\right|$$

with the critical value $t_{\alpha }=n-\log\left(1/\alpha \right)-1$, α∈(0,1), see [20,21]. So, there is no need to use the density distribution formula and it greatly simplifies the use of the test and makes it possible to use this test for any data compressor *f*.

It is important to note that there are tests developed within the AIT that can be used to test RNG [22,23].

#### 2.2.2. Test Performance Comparison

For test *T*, let us define the set $R_{T}$ of all infinite sequences that are random for *T*.

We use this definition to compare the “effectiveness” of different tests as follows. The test $T_{1}$ is more efficient than $T_{2}$ if the size of the difference $R_{T_{2}}\setminus R_{T_{1}}$ is not equal to zero, where the size is measured by the Hausdorff dimension.

Informally, the “smallest” set of random sequences corresponds to a test based on Kolmogorov complexity (4) (corresponding set $R_{K}$ contains “truly” random sequences). For a given test $T_{1}$ we cannot calculate the difference $R_{T_{1}}\setminus R_{K}$ because the statistic (4) is noncomputabele, but in the case of two tests $T_{1}$ and $T_{2}$, where $\dim(R_{T_{2}}\setminus R_{T_{1}})>0$, we can say that the set of sequences random according to $T_{2}$ contains clearly non-random sequences. So, in some sense, $T_{1}$ is more efficient than $T_{2}$. (Recall that we only consider computable tests.)

The definition of the Hausdorff dimension is given in the Appendix A, but here we briefly note that we use the Hausdorff dimension for it as follows: for any binary sequence $x_{1}x_{2}\ldots$ we define a real number σ(x)=0.$x_{1}x_{2}\ldots$ and for any set of infinite binary sequences *S* we denote the Hausdorff dimension of σ(S) by dimS. So, a test $T_{1}$ is more efficient than $T_{2}$ (formally $T_{1}⪰T_{2}$) if $\dim(R_{T_{2}}\setminus R_{T_{1}})>0$. Obviously, information about a test’s effectiveness can be useful to developers of the test’s batteries.

Also note that the Hausdorff dimension is widely used in information theory. Perhaps the first such use was due to Eggleston [24] (see also [25,26]), and later the Hausdorff dimension found numerous applications in AIT [27,28,29].

#### 2.2.3. Shannon Entropy

In RNG testing, one of the popular alternative hypotheses ($H_{1}$) is that the considered sequence generated by Markov process of memory (or connectivity) m,m>0, $\left(S_{m}\right)$, but the transition probabilities are unknown. ($S_{0}$, i.e., m=0, corresponds to the Bernoulli process). Another popular and perhaps the most general $H_{1}$ is that the sequence is generated by a stationary ergodic process ($S_{\infty }$) (excluding $H_{0}$).

Let us consider the Bernoulli process $\mu \in S_{0}$ for which μ(0)=p,μ(1)=q,(p+q=1). By definition, the Shannon entropy h(μ) of this process is defined as h(μ)=−(plogp+qlogq) [30]. For any stationary ergodic process ν∈S the entropy of order *k* is defined as follows:

$$h_{k}\left(\nu \right)=E_{\nu }\left(-\sum_{u\in {\left\{0,1\right\}}^{k}}\left(\nu \left(0/u\right)\log\left(0/u\right)+\nu \left(1/u\right)\log\nu \left(1/u\right)\right)\right),$$

where $E_{\nu }$ is the mathematical expectation according to ν, ν(z/u) is the conditional probability $\nu (x_{i+1}=z|x_{i-k}\ldots x_{i}=u)$, it does not depend on *i* due to stationarity [30].

It is known in Information Theory that for stationary ergodic processes (including $S_{\infty }$ and $S_{m},m\geq 0)$ $h_{k}\geq h_{k+1}$ for k≥0 and there exists the limit Shannon entropy $h_{\infty }\left(\nu \right)=\lim h_{k}\left(\nu \right)$. Besides, for $\nu \in S_{m}$$\;\;\;h_{\infty }=h_{m}$ [30].

Shannon entropy plays an important role in data compression because for any lossless and prefix-free code, the average codeword length (per letter) is at least as large as the entropy, and this limit can be reached. More precisely, let ϕ be a lossless, prefix-free code defined on ${\left\{0,1\right\}}^{n},n>0$, and let ν∈S. Then, for any ϕ, ν, and codewords of average length

(8) $$E_{n}\left(\phi ,\nu \right)=\frac{1}{n}\sum_{u\in {\left\{0,1\right\}}^{n}}\nu \left(u\right)\left|\phi \left(u\right)\right|$$

$E_{n}\left(\phi ,\nu \right)\geq h\left(\nu \right)$. In addition, there are codes $\phi_{1},\phi_{2},\ldots$ such that $\lim_{n\to \infty }E_{n}\left(\phi_{n},\nu \right)=h\left(\nu \right)$ [30].

#### 2.2.4. Typical Sequences and Universal Codes

The sequence $x_{1}x_{2}\ldots$ is typical for the measure $\mu \in S_{\infty }$ if for any word $y_{1}\ldots y_{r}$$\lim_{t\to \infty }N_{x_{1}\ldots x_{t}}\left(y_{1}\ldots y_{r}\right)/t=\mu \left(u\right)$, where $N_{x_{1}\ldots x_{t}}\left(y_{1}\ldots y_{r}\right)$ is the number of occurrences of a word $y_{1}\ldots y_{r}$ in a word $x_{1}\ldots x_{t}$.

Let us denote the set of all typical sequences as $T_{\mu }$ and note that $\mu (T_{\mu })=1$ [30]. This notion is deeply related to information theory. Thus, Eggleston proved the equality $\dim T_{\mu }=h\left(\mu \right)$ for Bernoulli processes ($\mu \in S_{0}$) [24], and later this was generalized for $\mu \in S_{\infty }$ [26,28].

By definition, a code ϕ is universal for a set of processes *S* if for any μ∈S and any $x\in T_{\mu }$

(9) $$\lim_{n\to \infty }\left|\phi \left(x_{1}\ldots x_{n}\right)\right|/n=h_{\infty }\left(\mu \right).$$

In 1968, R. Krichevsky [31] proposed a code $\kappa_{m}^{t}\left(x_{1}\ldots x_{t}\right)$, m≥0,t is an integer, whose redundancy, i.e., the average difference between the code length and Shannon entropy, is asymptotically minimal. This code and its generalisations are described in the Appendix A, but here we note the following main property. For any stationary ergodic process μ, that is, $\mu \in S_{\infty }$ and typical $x\in T_{\mu }$,

(10) $$\lim_{t\to \infty }\left|\kappa_{m}^{t}\left(x_{1}\ldots x_{t}\right)\right|/t=h_{m}\left(\mu \right)\;,$$

see [32].

Currently there are many universal codes which are based on different ideas and approaches, among which we note the PPM universal code [33], the arithmetic code [34], the Burrows–Wheeler transform [35], which is used along with the book-stack (or MTF) code [36,37,38], and some others [39,40,41].

The most interesting for us is the class of grammar-based codes suggested by Kieffer and Yang [7,42] which includes the Lempel–Ziv (LZ) codes [6] (note that perhaps the first grammar-based code was described in [43]).

The point is that all of them are universal codes and hence they “compress” stationary processes asymptotically to entropy and therefore cannot be distinguishable at $S_{\infty }$. On the other hand, we show that grammar-based codes can distinguish “large” sets of sequences as non-random beyond $S_{\infty }$.

#### 2.2.5. Two-Faced Processes

The so-called two-faced processes are described in [20,21] and their definitions will be given in Appendix A. Here, we note some of their properties: the set of two-faced processes $\Lambda_{s}\left(p\right)$ of order s,s≥1, and probability p,p∈(0,1), contains the measures λ from $S_{s}$ such that

$$h_{0}\left(\lambda \right)=h_{1}\left(\lambda \right)=\ldots =h_{s-1}\left(\lambda \right)=1,$$

(11) $$h_{s}\left(\lambda \right)=h_{\infty }\left(\lambda \right)=-\left(p\log p+\left(1-p\right)\log\left(1-p\right)\right).$$

Note that they are called two-faced because they appear to be truly random if we look at word frequencies whose length is less than *s*, but are “completely” non-random if the word length is equal to or greater than *s* (and *p* is far from 1/2).

## 3. Comparison of the Efficiency of Tests for Markov Processes with Different Memories and General Stationary Processes

We now describe the statistical tests for Markov processes and stationary ergodic processes as follows. By (6), statistical definitions are as follows:

$$\tau_{K_{m}^{t}}\left(x_{1}\ldots x_{n}\right)=n-\left|{\hat{\kappa }}_{m}^{t}\left(x_{1}\ldots x_{n}\right)\right|,$$

$$\tau_{R^{t}}\left(x_{1}\ldots x_{n}\right)=n-\left|{\hat{\rho }}^{t}\left(x_{1}\ldots x_{n}\right)\right|$$

where ${\hat{\kappa }}_{m}^{t}$ and ${\hat{\rho }}^{t}$ are universal codes for $S_{m}$ and $S_{\infty }$ defined in the Appendix A, see (A4) and (A5). We also denote the corresponding tests by $T_{K_{m}}^{t}$ and $T_{R}^{t}$. The following statement compares the performance of these tests.

**Theorem** **1.** *For any integers m,s and t=ms*

$$T_{K_{m}}^{t}⪯T_{K_{m+1}}^{t},\;\;T_{K_{m}}^{t}⪯T_{K_{R}}^{t}.$$

Moreover, $\dim(T_{K_{m}}^{t}\setminus T_{K_{m+1}}^{t})=1$.

**Proof.**  First, let us say a few words about the scheme of the proof. If we apply the $T_{K_{m}}^{t}$ test to typical sequences of a two-faced process $\lambda \in T_{\Lambda_{m+1(p)}},$ p≠1/2, they will appear random since $h_{m}\left(\lambda \right)=1$. So, the set of random sequences $R_{T_{K_{m}}^{t}}$ (i.e., random sequences according to $T_{K_{m}}^{t}$ test) contains the set of the typical sequences $T_{\Lambda_{m+1(p)}}$ for which $\dim(T_{\Lambda_{m+1(p)}})$ equals the limit Shannon entropy −(plogp+(1−p)log(1−p)). Hence, $\dim(R_{T_{K_{m}}^{t}})\geq$$\dim(T_{\Lambda_{m+1(p)}})=$−(plogp+(1−p)log(1−p)).

On the other hand, typical sequences of a two-faced process $\lambda \in T_{\Lambda_{m+1(p)}},p\neq 1/2$ are not random according to $T_{K_{m+1}}^{t}$ since $h_{m+1}\left(\lambda \right)$=−(plogp+(1−p)log(1−p))<1 (11) and the test. $T_{K_{m}}^{t}$ “compresses” them till the Shannon entropy −(plogp+(1−p)log(1−p)). So, $\dim(R_{T_{K_{m}}^{t}}\setminus R_{T_{K_{m+1}}^{t}})\geq$$\dim(R_{T_{K_{m}}^{t}})$≥−(plogp+(1−p)log(1−p)). Then $\sup_{p\in (0,1/2)}$$\dim(T_{K_{m}}^{t}\setminus T_{K_{m+1}}^{t})=1$.

More formally, consider a typical sequence *x* of $T_{\Lambda_{m+1(p)}}$, p≠1/2. So, $\lim_{t\to \infty }-\sum_{u\in {\left\{0,1\right\}}^{m+1}}$$\left(N_{x_{1}\ldots x_{t}}\left(u\right)/t\right)\log\left(N_{x_{1}\ldots x_{t}}\left(u\right)/t\right)=h_{\lambda }\left(m\right)=1,$ see (11), where the first equality is due to typicality, and the second to the property of two-faced processes (11).

From here and (A1), (A4) we obtain $E_{\lambda }\left(1/n\right)\left|{\hat{\kappa }}_{m}^{t}\left(x_{1}\ldots x_{n}\right)\right|$=1+ϵ, where ϵ>0. From this and typicality we can see that $\lim_{n\to \infty }\left|{\hat{\kappa }}_{m}^{t}\left(x_{1}\ldots x_{n}\right)\right|/n$ =1+ϵ. Hence, there exists such $n_{\delta }$ that $1+\epsilon -\delta <|{\hat{\kappa }}_{m}^{t}\left(x_{1}\ldots x_{n}\right)|/n<1+\epsilon +\delta$, if $n>n_{\delta }$. So $n-|{\hat{\kappa }}_{m}^{t}\left(x_{1}\ldots x_{n}\right)|$≤n−(n+ϵ−δ). So, if we take δ=ϵ/2, we can see that for $n>n_{\delta }$$n-|{\hat{\kappa }}_{m}^{t}\left(x_{1}\ldots x_{n}\right)|$ is negative. From this and the definition of randomness (5), we can see that typical sequences from $T_{\Lambda_{m+1(p)}}$ are random according to ${\hat{\kappa }}_{m}^{t}\left(x_{1}\ldots x_{n}\right)$, i.e., $T_{K_{m}}^{t}$. From this and (A6), we obtain $T_{K_{m+1}}^{t}⪯T_{R}^{t}$. □

## 4. Effectiveness of Tests Based on Lempel-Ziv Data Compressors

In this part we will describe a test that is more effective than $T_{R}^{t}$ and $T_{K_{m}}^{t}$ for any *m*.

First, we will briefly describe the LZ77 code based on the definition in [44]. Suppose there is a binary string $\sigma^{*}$ that is encoded using the code LZ77. This string is represented by a list of pairs $\left(p_{1};l_{1}\right)\ldots \left(p_{s};l_{s}\right)$. Each pair $\left(p_{i};l_{i}\right)$ represents a string, and the concatenation of these strings is $\sigma^{*}$. In particular, if $p_{i}=0$, then the pair represents the string $l_{i}$, which is a single terminal. If $p_{i}\neq 0$, then the pair represents a portion of the prefix of $\sigma^{*}$ that is represented by the preceding i−1 pairs; namely, the $l_{i}$ terminals beginning at position $p_{i}$ in $\sigma^{*}$; see ([44] part 3.1). The length of the codeword depends on the encoding of the sub-words $p_{i},l_{i}$ which are integers. For this purpose we will use a prefix code *C* for integers, for which for any integer *m*

(12) |C(m)|=logm+2loglog(m+1)+O(1).

Such codes are known in information theory; see, for example, ([30] part 7.2). Note that *C* is the prefix code and, hence, for any r≥1 the codeword $C\left(p_{1}\right)C\left(l_{1}\right)\ldots C\left(p_{r}\right)C\left(l_{r}\right)$ can be decoded to $\left(p_{1};l_{1}\right)\ldots \left(p_{r};l_{r}\right)$. There is the following upper bound for the length of the LZ77 code [30,44]: for any word $w_{1}w_{2}\ldots .w_{m}$

(13) $$|code_{LZ}\left(w_{1}w_{2}\ldots w_{m}\right)|\leq m\;\left(1+o\left(1\right)\right),$$

if m→∞.

We will now describe such sequences that, on the one hand, are not typical for any stationary ergodic measure and, on the other hand, are not random and will be rejected by the suggested test. Thus, the proposed model allows us to detect non-random sequences that are not typical for for any stationary processes.On the other hand, those sequences are recognized tests based on LZ77 as non-random. To do this, we take any random sequence $x=x_{1}x_{2}\ldots$ (that is, for which (4) is valid) and define a new sequence $y\left(x\right)=y_{1}y_{2}\ldots$ as follows. Let for k=0,1,2,…

$$u_{k}=x_{2^{2^{k}}-1}x_{2^{2^{k}}}x_{2^{2^{k}}+1}\ldots x_{2^{2^{k+1}}-2}$$

(14) $$y\left(x\right)=u_{0}u_{0}u_{1}u_{1}u_{2}u_{2}u_{3}u_{3}\ldots$$

For example, $u_{0}=x_{1}x_{2}$, $u_{1}=x_{3}$$x_{4}$…$x_{14}$, $u_{2}=x_{15}\ldots x_{254}$, $y\left(x\right)=x_{1}x_{2}$$x_{1}x_{2}x_{3}x_{4}\ldots x_{14}$$x_{3}x_{4}\ldots x_{14}$ $x_{15}\ldots x_{254}$$x_{15}\ldots x_{254}$….

The idea behind this sequence is quite clear. Firstly, it is obvious that the word *y* cannot be typical for a stationary ergodic source and, secondly, when $u_{0}u_{0}u_{1}u_{1}\ldots u_{k}u_{k}$ is encoded the second subword $u_{k}$ will be encoded by a very short word (about $O(\log|u_{k}|))$, since it coincides with the previous word $u_{k}$. So, for large *k* the length of the encoded word $LZ(u_{0}u_{0}u_{1}u_{1}\ldots u_{k}u_{k})$ will be about $|u_{0}u_{0}u_{1}u_{1}\ldots u_{k}u_{k}|\;\left(1/2+o\left(1\right)\right)\;$. So, $\lim\inf_{n\to \infty }\left|LZ\left(y_{1}y_{2}\ldots y_{n}\right)\right|/n=1/2$. Hence, it follows that

(15) dim({y(x):xisrandom})=1/2.

Here, we took into account that *x* is random and, dim{x:xisrandom}=1, see [28].) So, having taken into account the definitions of non-randomness (6) and (7), we can see that y(x) is non random according to statistics $\tau =n-|LZ(y_{1}\ldots y_{n})|$. Denote this test by $T_{LZ}$.

Let us consider the test $T_{K_{m}}^{t}$, m,t are integers. Having taken into account that the sequence *x* is random, we can see that $\lim_{t\to \infty }$$|\kappa_{m}^{t}\left(x_{i}x_{i+1}\ldots x_{i+t}\right|/t=1.$ So, from from (A4) we can see that for any *n* $|{\hat{\kappa }}_{m}^{t}\left(x_{1}\ldots x_{n}\right|/t=1+o\left(1\right)$. The same reasoning is true for the code ${\hat{\rho }}^{t}$.

We can now compare the size of random sequence sets across different tests as follows:

$$R_{T_{K_{m}}^{t}}\setminus R_{T_{LZ}}⊃\left\{y\left(x\right):x\;is\;random\right\}\;.$$

Taking into account (15), we can see that

$$\dim(R_{T_{K_{m}}^{t}}\setminus R_{T_{LZ}})\geq 1/2\;.$$

Likewise, the same is true for the $T_{R}$ test. From the latest inequality we obtain the following.

**Theorem** **2.** *For any random (according to (4)) sequence x the sequence y(x) is non-random for the test $T_{LZ}$, whereas this sequence is random for tests $T_{R}^{t}$ and $T_{K_{m}}^{t}$. Moreover, $T_{R}^{t}⪯T_{LZ}$ and $T_{K_{m}}^{t}⪯T_{LZ}$ for any m,t.*

**Comment.** The sequence y(x) is constructed by duplicating parts of *x*. This construction can be slightly modified as follows: instead of duplication (as $u_{i}u_{i}$), we can use $u_{i}u_{i}^{\gamma }$, where $u_{i}^{\gamma }$ contains γ|u| the first letters of *u*, γ<1/2. In this case, $\dim(R_{T_{K_{m}}^{t}}\setminus R_{T_{LZ}})\geq 1-\gamma \;$ and, therefore,

$$\underset{\gamma \in (0,1/2)}{\sup}\dim\left(R_{T_{K_{m}}^{t}}\setminus R_{T_{LZ}}\right)=1\;.$$

## 5. Conclusions

Here, we describe some recommendations for the practical testing of RNGs, based on the method of comparing the power of different statistical tests. Based on Theorem 1, we can recommend to use several tests $T_{K_{s}^{t}}$, based on the analysis of occurrence frequencies of words of different length *s*. In addition, we recommend using tests for which *s* depends on the length *n* of the sequence under consideration. For example, $s_{1}=O\left(\log\log n\right)$), $s_{2}=O(\sqrt{\log n}$), etc. They can be included in the test battery directly or as the “mixture” $T_{R}$ with several non-zero β coefficients, see (A2) in the Appendix A.

Theorem 2 shows that it is useful to include tests based on dictionary data compressors such as the Lempel–Ziv code. In such a case we can use the statistic

$$\tau_{LZ}=n-\left|LZ\left(y_{1}\ldots y_{n}\right)\right|$$

with the critical value $t_{\alpha }=n-\log\left(1/\alpha \right)-1$, α∈(0,1), see [20,21]. Note that in this case, there is no need to use the density distribution formula, which greatly simplifies the use of the test and makes it possible to use a similar test for any grammar-based data compressor.

## Data Availability

Data is contained within the article.

## Appendix A

### Appendix A.1. Hausdorff Dimension

Let A⊂[0,1],ρ>0. A family of sets *S* is called a ρ-covering *A* if

(i) *S* is finite or countable, (ii) any σ⊂[0,1] and its length is not greater than ρ and (iii) $\cup_{\sigma \in S}\sigma ⊃A$. Let

$$l\left(\alpha ,A,\rho \right)=\inf\sum_{\sigma \in S}diam{\left(\sigma \right)}^{\alpha },$$

where the infimum is taken over all ρ-coverings. Then, Hausdorff dimension dim(A) is determined by the equality

$$\dim\left(a\right)=\underset{\alpha }{\inf}\lim_{\rho \to 0}l\left(\alpha ,A,\rho \right)=0=\underset{\alpha }{\sup}\lim_{\rho \to 0}l\left(\alpha ,A,\rho \right)=\infty .$$

### Appendix A.2. Krichevsky Universal Code and Twice-Universal Code

Krichevsky in [31] described the following measure $K_{0}$ and universal code $\kappa_{0}$ for Bernoulli processes, which in the case of the binary alphabet looks like

$$K_{0}^{t}\left(x_{1}x_{2}\ldots x_{t}\right)=\prod_{i=0}^{t-1}\frac{N_{x_{1}\ldots x_{i}}\left(x_{i+1}\right)+1/2}{i+1},$$

$$\kappa_{0}^{t}\left(x_{1}x_{2}\ldots x_{t}\right)=\left\lceil -\log K_{0}\left(x_{1}x_{2}\ldots x_{t}\right)\right\rceil \;.$$

Then, he generalized them for Markov chains of memory m,m>0 [32], as follows:

$$K_{m}^{t}\left(x_{1}\ldots x_{t}\right)=\left\{\begin{array}{cc} \frac{1}{2^{t}} & \\ \;\;\;\;\mathrm{if}\;t\leq \;m\\ \frac{1}{2^{t}}\prod_{i=m}^{t-1}\frac{N_{x_{1}\ldots x_{i}}\left(x_{i+1-m}\ldots x_{i+1}\right)+1/2}{N_{x_{1}\ldots x_{i-1}}\left(x_{i+1-m}\ldots x_{i}\right)+1} & \\ \;\;\;\;\mathrm{if}\;t>m, \end{array}\right.$$

$\kappa_{m}^{t}\left(x_{1}\ldots x_{t}\right)=\left\lceil -\log K_{m}^{t}\left(x_{1}\ldots x_{t}\right)\right\rceil$, see [32]. For example,

$$K_{0}^{5}\left(01010\right)=\frac{1/2}{1}\frac{1/2}{2}\frac{132}{3}\frac{3/2}{4}\frac{5/2}{5},$$

$$K_{1}^{5}\left(01010\right)=\frac{1}{2}\frac{1/2}{1}\frac{1/2}{1}\frac{3/2}{2}\frac{3/2}{2}\;.$$

The code $\kappa_{m}^{t}$ is universal for a set of processes $S_{m}$, and, for any $\nu \in S_{m}$

(A1) $$h_{m}\left(\nu \right)<E_{\nu }\left(\kappa_{m}^{t},\nu \right)\leq h_{m}\left(\nu \right)+2^{m}\log t/\left(2t\right)+O\left(1/t\right)$$

Refs. [31,32]. (This code is optimal in the sense that the redundancy, that is $2^{m}\log t/\left(2t\right)+\left(1/t\right)$, is asymptotically minimal [31,32].)

One of the first universal codes for the set of all stationary ergodic processes $S_{\infty }$ was proposed in [45]. For this code, the measure ρ and the code length *R* are defined as follows:

(A2) $$R^{t}\left(x_{1}\ldots x_{t}\right)=\sum_{i=0}^{\infty }\beta_{i}K_{i}^{t}\left(x_{1}x_{2}\ldots x_{t}\right),$$

$$\rho^{t}\left(x_{1}\ldots x_{t}\right)=\left\lceil -\log R^{t}\left(x_{1}x_{2}\ldots x_{t}\right)\right\rceil \;,$$

where $\sum_{i=0}^{\infty }\beta_{i}=1$ and $\forall i:\beta_{i}>0$. Obviously, for any *j*

$$-\log\sum_{i=0}^{\infty }\beta_{i}K_{i}^{t}\left(x_{1}x_{2}\ldots x_{t}\right)=-\log\beta_{j}K_{j}^{t}\left(x_{1}x_{2}\ldots x_{t}\right)+$$

$$-\log(1+\sum_{i=0,i\neq j}^{\infty }\beta_{i}K_{i}^{t}\left(x_{1}x_{2}\ldots x_{t}\right)/\left(\beta_{j}K_{j}^{t}\left(x_{1}x_{2}\ldots x_{t}\right)\right)$$

$$\leq -\log\beta_{j}K_{j}^{t}\left(x_{1}x_{2}\ldots x_{t}\right)\;.$$

Hence,

$$\rho^{t}\left(x_{1}\ldots x_{t}\right)\leq \left\lceil -\log\beta_{j}-\log K_{j}^{t}\left(x_{1}x_{2}\ldots x_{t}\right)\right\rceil \leq \left\lceil -\log\beta_{j}\right\rceil$$

(A3) $$+\left\lceil -\log K_{j}^{t}\left(x_{1}x_{2}\ldots x_{t}\right)\right\rceil =\left\lceil -\log\beta_{j}\right\rceil +\left|\kappa_{j}^{t}\left(x_{1}x_{2}\ldots x_{t}\right)\right|.$$

This code is called twice universal [45] because it can be used to compress data when both the process memory and the probability distribution are unknown.

Usually, when using universal codes, the sequence $x_{1}\ldots x_{n}$ is encoded in parts as follows:

(A4) $${\hat{\kappa }}_{m}^{t}\left(x_{1}\ldots x_{n}\right)=\kappa_{m}^{t}\left(x_{1}\ldots x_{t}\right)\kappa_{m}^{t}\left(x_{t+1}\ldots x_{2t}\right)\ldots .\kappa_{m}^{t}\left(x_{n-t+1}\ldots x_{n}\right)$$

(for brevity, we assume that n/t is an integer). Let us similarly define

(A5) $${\hat{\rho }}^{t}\left(x_{1}\ldots x_{n}\right)=\rho^{t}\left(x_{1}\ldots x_{t}\right)\rho^{t}\left(x_{t+1}\ldots x_{2t}\right)\ldots .\rho^{t}\left(x_{n-t+1}\ldots x_{n}\right)$$

Taking into account the definition of $\kappa_{j}^{t}\left(x_{1}x_{2}\ldots x_{t}\right)$ and Equations (4), (A4) and (A5) we obtan that for any integer *j*

(A6) $${\hat{\rho }}^{t}\left(x_{1}\ldots x_{n}\right)\leq {\hat{\kappa }}_{j}^{t}\left(x_{1}\ldots x_{n}\right)+O\left(n/t\right).$$

### Appendix A.3. Two-Faced Processes

Let us first consider several examples of two-faced Markov chains. Let a matrix of transition probabilities $T_{1}$ be as follows:

,

where ν∈(0,1) (i.e., $P\{x_{i+1}=0|x_{i}=0\}=\nu$, $P\{x_{i+1}=0|x_{i}=1\}=1-\nu ,\ldots$). The “typical” sequences for ν=0.9 and ν=0.1 can be as follows:

0000000000111111111000000000011111110…,

0101010110101010100101010101010101011010….

(Here, the gaps correspond to state transitions.) Of course, these sequences are not truly random. On the other hand, the frequencies of 1s and 0s go to 1/2 due to the symmetry of the matrix $T_{1}$.

Define

(Here $\;\;P\{x_{i+1}=0|x_{i}=0,x_{i-1}=0\}=\nu$, $P\{x_{i+1}=0|x_{i}=0,x_{i-1}=1\}=1-\nu ,\ldots \;$.)

Now, we can define a transition matrix with two-faced Markov chains with different memory as follows.

The k+1-order transition matrix $T_{k+1}=T_{k}\hat{T_{k}}$, ${\hat{T}}_{k+1}=\hat{T_{k}}T_{k}$, k=2,3,…. T In order to define the process $x_{1}x_{2}\ldots$ the initial probability distribution needs to be specified. We define the initial distribution of the processes $T_{k}$ and ${\bar{T}}_{k}$, k=1,2,…, to be uniform on ${\left\{0,1\right\}}^{k}$, i.e., $P\left\{x_{1}\ldots x_{k}=u\right\}=2^{-k}$ for any $u\in {\left\{0,1\right\}}^{k}$.

The following statement from [20,21] describes the main properties of the processes defined above.

**Claim.** Let a sequence $x_{1}x_{2}\ldots$ be generated by the process $T_{k}$ (or ${\bar{T}}_{k}$), k≥1 and *u* be a binary word of length *k*. Then, if the initial state obeys the uniform distribution over ${\left\{0,1\right\}}^{k}$, then

(i) For any j≥0
  (A7) $$P\left(x_{j+1}\ldots x_{j+k}=u\right)=2^{-|u|}.$$
(ii) For each ν∈(0,1), the *k*-order Shannon entropy ($h_{k}$) of the processes $T_{k}$ and ${\bar{T}}_{k},$ equals 1 bit per letter, whereas the limit Shannon entropy ($h_{\infty }$) equals $-(\nu \log_{2}\nu +\left(1-\nu \right)\log_{2}\left(1-\nu \right)).$
